# Song Processing in the Zebra Finch Auditory Forebrain Reflects Asymmetric Sensitivity to Temporal and Spectral Structure

**DOI:** 10.3389/fnins.2017.00549

**Published:** 2017-10-05

**Authors:** Lisbeth Van Ruijssevelt, Stuart D. Washington, Julie Hamaide, Marleen Verhoye, Georgios A. Keliris, Annemie Van der Linden

**Affiliations:** Bio-Imaging Lab, Department of Biomedical Sciences, University of Antwerp, Antwerp, Belgium

**Keywords:** hemispheric lateralization, zebra finch, songbird, functional MRI, auditory processing, spectro-temporal

## Abstract

Despite being commonly referenced throughout neuroscientific research on songbirds, reports of hemispheric specialization in the processing of song remain controversial. The notion of such asymmetries in songbirds is further complicated by evidence that both cerebral hemispheres in humans may be specialized for different aspects of speech perception. Some studies suggest that the auditory neural substrates in the left and right hemispheres of humans process temporal and spectral elements within speech sounds, respectively. To determine whether songbirds process their conspecific songs in such a complementary, bilateral manner, we performed functional magnetic resonance imaging (fMRI) on 15 isoflurane anesthetized adult male zebra finches (*Taeniopygia guttata*) while presenting them with (1) non-manipulated, (2) spectrally-filtered (reduced spectral structure), and (3) temporally-filtered (reduced temporal structure) conspecific song. Our results revealed sensitivity of both primary (Field L) and secondary (caudomedial nidopallium, NCM) auditory regions to changes in spectral and temporal structure of song. On the one hand, temporally-filtered song elicited a bilateral decrease in neural responses compared to the other stimulus types. On the other hand, spectrally filtered song elicited significantly greater responses in left Field L and NCM than temporally filtered or non-manipulated song while concurrently reducing the response relative to non-manipulated song in the right auditory forebrain. The latter hemispheric difference in sensitivity to manipulations of spectral structure in song, suggests that there is an asymmetry in spectral and temporal domain processing in the zebra finch auditory forebrain bearing some resemblance to what has been observed in human auditory cortex.

## Introduction

The contributions of the left and right cerebral hemispheres to the processing of human speech remain controversial. The classical view is that the left hemisphere contains a uniquely human closed system (i.e., a speech module) for processing speech and other linguistic stimuli with little to no contribution from the right hemisphere (Liberman and Mattingly, 1989). However, increasing evidence suggests that the left and right hemispheres provide differential yet complementary contributions to speech perception. One possibility is that the left and right auditory cortices are, respectively, specialized for temporal and spectral domain processing (Zatorre et al., 2002; Poeppel, 2003). Since time and frequency are canonically conjugate variables (Gabor, 1947; Joos, 1948), a necessary consequence of this specialization is that at the upper limit of resolution the refined temporal precision of the left auditory cortex (e.g., the ability to process rapid changes in frequency, such as the formant transitions found in consonants) must come at the expense of spectral precision (Robin et al., 1990; Zatorre and Belin, 2001). Likewise, the refined spectral precision (e.g., the ability to detect variations in prosody and speaker identity) of the right auditory cortex has been demonstrated by these same studies to come at the expense of temporal precision (Robin et al., 1990; Zatorre and Belin, 2001). Such a domain-general perspective implies that animals other than humans may possess comparable hemispheric specializations for processing their conspecific communication sounds. Other than the spectral vs. temporal hypothesis for left hemispheric specialization for speech perception described above, alternative possibilities include functional specialization of somatosensory and motor areas determining left hemispheric specialization for speech perception within auditory processing streams (Liebenthal et al., 2013) and speech-selective acoustic sensitivities in the left hemisphere that are indissociable from the articulatory systems that produce speech sounds (McGettigan and Scott, 2012).

Songbirds are the most common and best-known animal models of human speech, owing to their ability to learn their songs from tutors and the critical periods associated with this vocal learning (Doupe and Kuhl, 1999; Brainard and Doupe, 2002). The songs of certain bird species (e.g., zebra finches) are highly stereotyped and consist of a rich spectro-temporal structure, comparable to human speech sounds. It stands to reason that songbirds may process the temporal and spectral components of their complex conspecific songs in different cerebral hemispheres, as is postulated in humans. However, most studies of hemispheric differences in the processing of conspecific birdsong have used complete, non-manipulated songs rather than spectral and/or temporal song components. Results from these studies are less than consistent, with most of them implicating left hemispheric auditory forebrain nuclei as auditory (memory) centers supporting the acquisition, processing, and recognition of conspecific birdsong (Cynx et al., 1992; Avey et al., 2005; Hauber et al., 2007; Poirier et al., 2009; Moorman et al., 2012). Others show a right hemispheric specialization for conspecific birdsong perception (Voss et al., 2007; Phan and Vicario, 2010), suggest a hemispheric asymmetry in the processing of harmonic structure vs. familiarity of song (Cynx et al., 1992), or report no hemispheric differences in the perception of conspecific song at all (Chew et al., 1996). Showing hemispheric differences in temporal and spectral domain processing in the auditory forebrains of songbirds could shed light on this controversy and demonstrate that the observed asymmetry is a functional analog of the classically reported hemispheric specialization for speech perception in humans.

Here, we performed functional magnetic resonance imaging (fMRI) experiments on 15 adult male zebra finches while presenting them with three stimulus types: (a) non-manipulated, (b) spectrally filtered (spectral information reduced with temporal information retained), and (c) temporally filtered (temporal information reduced with spectral information retained) unfamiliar conspecific song. Voxel-based and region-of-interest (ROI) analyses revealed significantly differential sensitivity to manipulations of spectral and temporal structure of song in left vs. right hemispheric auditory forebrain nuclei.

## Materials and methods

### Subjects

We obtained 15 adult male zebra finches (*Taeniopygia guttata*, >100 days old) from a local breeder for use in this study. We housed the birds together in a large, same-sex aviary maintained under a 12 h light/dark cycle with access to water and food *ad libitum*. The ethical committee of the University of Antwerp approved all experimental procedures (License number: 2016-32), which were in agreement with the Belgian laws on the protection and welfare of animals.

### Auditory stimuli

**Figure 1:**
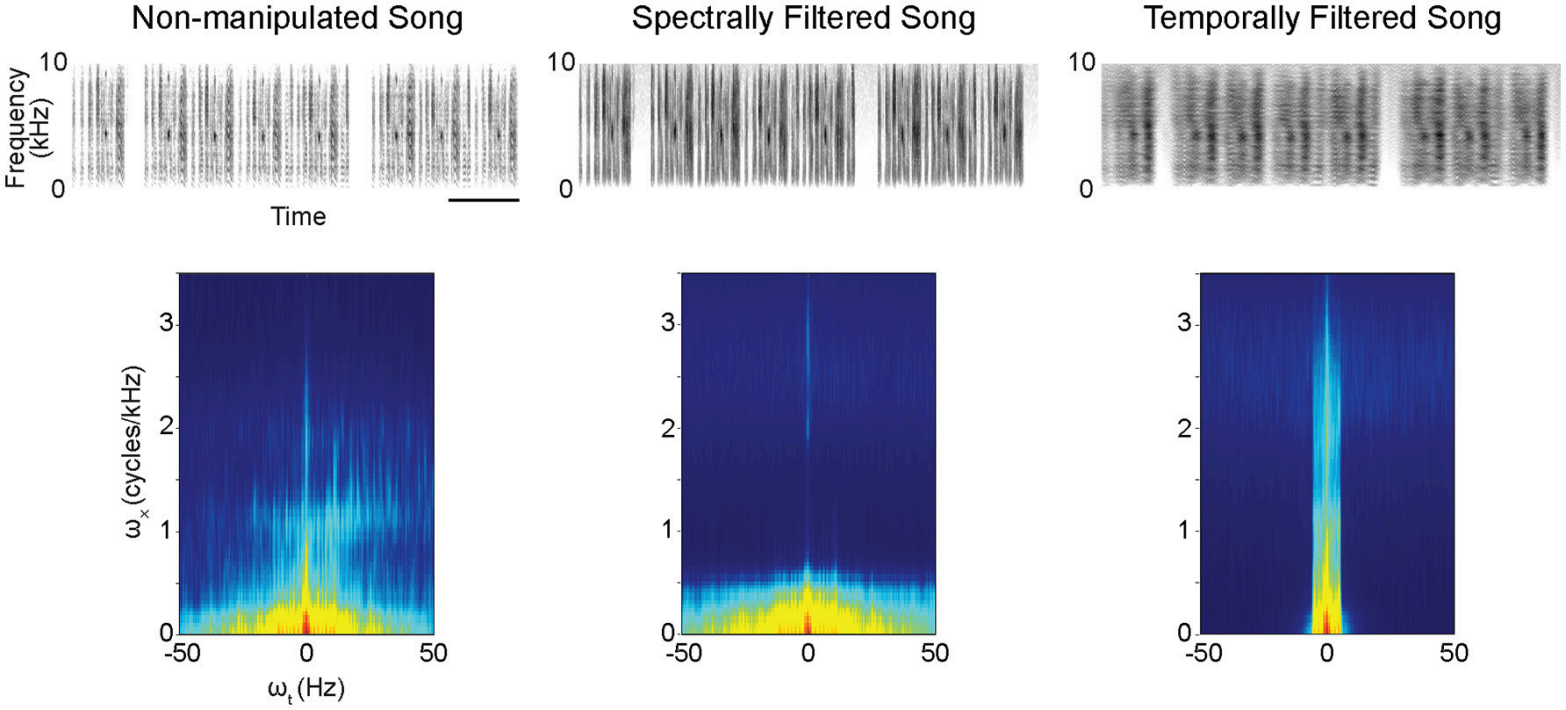
Example spectrograms (excerpts from original 16-s stimuli) **(Top)** and modulation power spectra **(Bottom)** for the different stimulus types. The modulation power spectra quantify the spectro-temporal structure present in the sounds. Filtering to reduce spectral or temporal information from the songs was performed on these modulation power spectra. ω_x_ = spectral modulations, ω_t_ = temporal modulations. scale bar = 1s.

Conspecific songs were selected from previous recordings of four different male zebra finches. Our subjects were not housed with, nor did they have any contact with, the four birds that produced these songs. From the recordings, we selected and concatenated multiple song bouts to create 16 s long stimuli for each male's song separately. Applying a MATLAB-based algorithm for spectral and temporal filtering (Singh and Theunissen, 2003, http://theunissen.berkeley.edu/Software.html) to the song stimuli from the four males, we generated three stimulus types containing songs with varying levels of temporal and spectral structure (Figure 1, Figure S1): (a) non-manipulated song consisting of the song bouts as recorded (Audios S1, S4, S7, S10), (b) spectrally filtered song (Audios S2, S5, S8, S11), and (c) temporally filtered song (Audios S3, S6, S9, S12). For both spectral and temporal filtering, a low pass filtering procedure was performed in the space of the modulation spectrum (Singh and Theunissen, 2003; Figure 1-bottom) as previously described (Boumans et al., 2007; Elliott and Theunissen, 2009). Initial spectrograms were made with 125-Hz frequency width Gaussian filters. These spectrograms were then log transformed after which a 2D fast Fourier transform was taken to calculate the modulation spectrum (i.e., joint spectral and temporal amplitude spectrum). The applied filtering procedure on these modulation spectra generated songs with equal power and overall frequency power spectra but with a reduction of either the natural temporal modulations or the natural spectral modulations. For spectral filtering, the spectral modulation frequency cut-off was set at 5 × 10^−4^ cycles/Hz, which means that spectral structure within any 2-kHz band was filtered out (i.e., pitch below 2 kHz is filtered out). For temporal filtering, the temporal modulation frequency cut-off was set at 5 Hz, which means that all amplitude envelope changes, that are faster than 5 Hz were filtered out. This differential filtering method thus did not remove all temporal and/or spectral information from a song but only reduced it. Note also that we cannot completely exclude the possibility that filtering of spectral features in a song did not influence temporal song features to some extent and vice-versa.

### Data acquisition and stimulation protocol

We acquired all of our MRI data on a horizontal 7 Tesla MR system (Pharmascan 70/16 US, Bruker Biospin, Germany) following established protocols (Van Ruijssevelt et al., 2013). In short, fMRI scans consisted of a time series of 298 T_2_-weighted rapid acquisition relaxation-enhanced (RARE) volumes of 15 slices covering the whole brain (slice thickness = 0.75 mm, interslice gap = 0.05 mm) with an in-plane resolution of (0.25 × 0.25) mm^2^. We acquired a high-resolution anatomical 3D RARE image [resolution = (70 × 70 × 70) μm^3^] for each bird in the same orientation as the fMRI scans to facilitate subsequent spatial registration of images. During the experiment, the animals were continuously anesthetized with 1.2% isoflurane in a mixture of oxygen and nitrogen (at flow rates of 100 and 200 cm^3^/min, respectively).

During the fMRI session, the birds were exposed to the three different stimulus types (non-manipulated song, spectrally filtered song, temporally filtered song) derived from a song from one of the four males (either song 1, 2, 3, or 4; respectively Audios S1, S4, S7, S10) as described above. Stimulus types were pseudo-randomly presented in an ON/OFF blocked paradigm that alternated of 16 s of stimulation (ON periods) and 16 s of rest (OFF period). Presentation of the three song types was always grouped together in consecutive ON blocks to ensure an equal number of presentations per stimulus type at the end of the scanning session. The order in which the three types were presented within each consecutive repetition of the group of stimuli was randomized (for example: unmanipulated (UN), spectrally filtered (SF), temporally filtered (TF), | SF, TF, UN, | UN, TF, SF, | SF, TF, UN | etc.). In total, an fMRI session consisted of 72 ON blocks (24 per stimulus type) and 72 OFF blocks. Two T_2_-weighted RARE images were acquired during each block, resulting in 48 images per stimulus type, per subject. Auditory stimuli were played back at a mean intensity (root-mean-square) of 70 dB through dynamic loudspeakers (Visation, Germany; magnets removed) placed at both sides of the bird's head. To ensure the validity of any observed hemispheric differences, the orientation of the headphones was interchanged between consecutive experiments (subject 1: left speaker 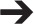 right ear; Subject 2: left speaker 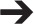 left ear; Subject 3: left speaker 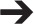 right ear; etc.).

### Data analysis

Our inclusion criteria for fMRI time series in the subsequent analysis were (1) limited head motion (<0.5 mm translation in either of the image's 3 directions), and (2) detection of bilateral positive BOLD responses in the primary auditory region Field L.

We performed both data preprocessing and statistical voxel-based analyses using the Statistical Parametric Mapping toolbox (SPM12, Wellcome Trust Centre for Neuroimaging, London, UK; http://www.fil.ion.ucl.ac.uk/spm). These analyses were similar to those previously described (Van Ruijssevelt et al., 2013). In short, we realigned and co-registered the fMRI time series to their corresponding 3D RARE. A population based template was generated from the anatomical 3D RARE images of all animals (ANTs; http://stnava.github.io/ANTs/). Spatial normalization of all scans to this template enabled between-subject comparisons (Avants et al., 2011). Finally, to achieve in-plane smoothing, we applied a Gaussian kernel of 0.5 mm full width at half maximum (FWHM).

High pass filtering (352-s cutoff period) removed low frequency drifts in the BOLD signal. For the first level analysis, we subsequently modeled for each subject the BOLD responses as a box-car function convolved with a canonical hemodynamic response function within the framework of the general linear model (GLM) to analyze brain activation differences related to the onset of the different stimuli. We included the six estimated movement parameters derived from the realignment corrections as regressors in the model to account for the residual effect of head motion and restricted the analysis to voxels within the brain by using a whole brain mask. After the estimation of the GLM parameters (β), we calculated different t-contrast images (containing weighted parameter estimates) for different comparisons including non-manipulated song vs. rest, temporally filtered song vs. rest and spectrally filtered song vs. rest as well as comparisons of the activations of these different stimulus types between each other.

Next, to study the effect of song filtering on the activation of auditory regions in the zebra finch forebrain at the group (second) level, we entered the subjects' contrast images (non-manipulated song vs. rest, temporally filtered song vs. rest and spectrally filtered song vs. rest) into a flexible factorial analysis with subjects as random variable. To explore the main effect of stimulus class, we restricted the analysis to voxels that showed a positive BOLD response to any song (*t*-test all stimuli vs. rest). Within the main effect of stimulus (*p*_*uncorrected*_ < 0.005), we performed *post-hoc t*-tests to determine regions sensitive to temporal and/or spectral filtering of song. Since we performed statistical tests on a voxel-by-voxel basis, *p*-values were adjusted to the number of independent tests performed via Family Wise Error (FWE) correction. As this is a voxel based analysis, results are reported by the highest voxel *t*-value within each cluster (*t*_max_) and the associated voxel *p*-value. Our threshold for significance was *p*_*FWE*_ < 0.05. For visualization, an explorative threshold of *p*_*uncorrected*_ < 0.005 was applied.

Further, to assess laterality of the observed sensitivity to spectral and temporal filtering in the auditory forebrain, we used the “AveLI” SPM add-on to compute lateralization indices (LIs) for the three song types for left vs. right regions of interest (ROIs) in a threshold-free manner (Matsuo et al., 2012; freely available at http://aveli.web.fc2.com/). The ROI for this analysis was determined by the ensemble of voxels (and their mirrored counterparts) in which a significant effect of song type was determined in the voxel-based group analysis (main effect of stimulus class; see results). The computed LI by the AveLI code represents the portion of the signal that is found either left (values toward −1) or right (values toward +1). Further, we verified the calculated AveLIs with alternative measures for calculating LI described in literature (Fernández et al., 2001; Jansen et al., 2006; Seghier, 2008), including LIs based on the number of significant voxels as well as on the magnitude of the effect in the left vs. the right hemisphere using either no threshold (Seghier, 2008) or a variable subject specific adjusted threshold (Fernández et al., 2001) to determine significant voxels. We found that the LIs obtained with the different methods correlated well (Figure S2) suggesting that our results were not significantly influenced by the method chosen to calculate LIs. We assessed LIs for different effects found to be significant in the voxel-based analysis: non-manipulated song > temporally filtered song, non-manipulated song > spectrally filtered song, spectrally filtered song > temporally filtered song, spectrally filtered song > non-manipulated song. These comparisons are further referred to as “filtering effects”. The LI indices were derived from *t*-contrast maps for these different filtering effects at the single subject level. Statistical analysis on the calculated LI-values was performed in JMP® (Version 13, SAS Institute Inc., Cary, NC, 1989–2007). First, we assessed whether for each filtering effect, the LI was significantly different from 0 using separate one-sample *t*-tests. Next, we compared the LIs of the different effects using a linear mixed model with filtering effect as a fixed, and subject as a random, variable. We used unbounded variance components to fit the data using the restricted maximum likelihood method. Tukey's HSD (honest significant difference) test was used for all *post-hoc* tests and results were considered significant for *p* < 0.05. Data are represented as mean ± standard error of the mean (SEM).

## Results

**Figure 2:**
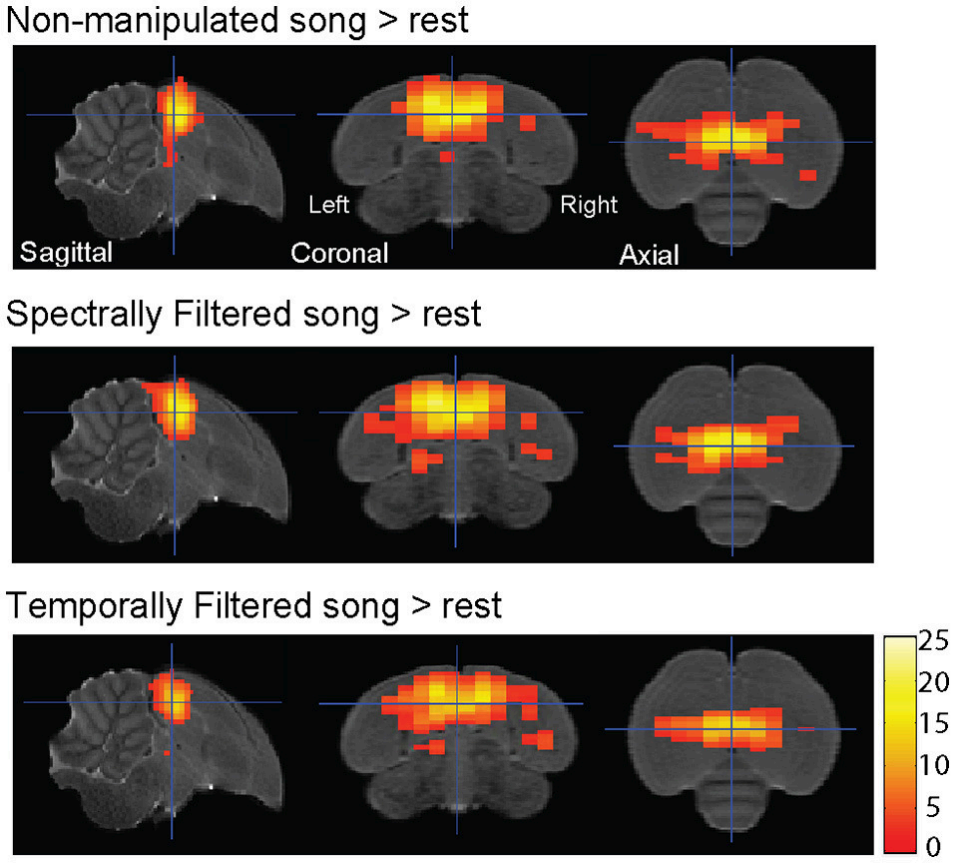
Auditory forebrain activation in response to the different stimuli. Statistical maps are superimposed on images from the population based template. Only voxels with *t* > 2.76 (one sample *t*-test, *p*_uncorrected_ < 0.005) are displayed (*n* = 15).

Voxel-based group analysis revealed extensive bilateral activation of the auditory lobule in response to all stimulus types vs. the rest periods (Figure 2).

**Figure 3:**
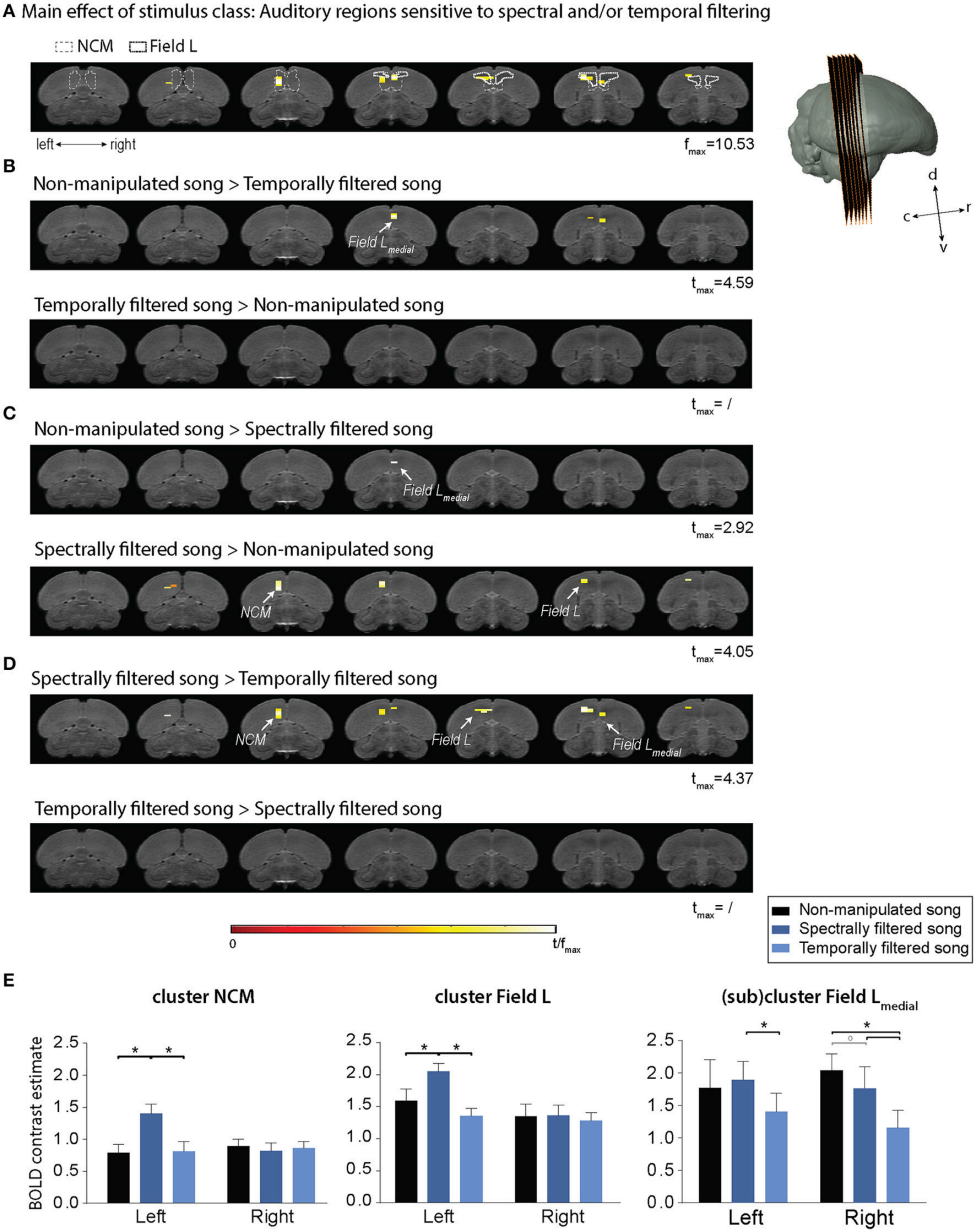
Effect of temporal and spectral filtering of song on neural activation of the auditory forebrain. The image series represents adjacent coronal slices of the population based template with the left image corresponding to the most caudal and the right to the most rostral slice as illustrated by the 3D rendering on the right. Statistical maps are superimposed on the image series. *T*-values are color coded according to the scale displayed in the figure. All voxels with *t* > 2.76 (*p*_uncorrected_ < 0.005) are displayed. **(A)** Statistical map of the main effect of stimulus class (one way ANOVA—within subjects). Delineations of two auditory forebrain regions from the zebra finch MRI atlas dataset as published earlier are included as a guide for the localization of the effect (Poirier et al., 2008; van der Kant et al., 2013). **(B–D)** Statistical maps of all *post-hoc t*-tests. **(E)** Average of the estimated relative response amplitude (BOLD contrast estimates (β), expressed in non-dimensional units) elicited by playback of non-manipulated song and its corresponding temporally and spectrally filtered variants in the indicated clusters (clusters of significant voxels in different sub-regions as illustrated in the maps above and mirrored counterparts in the opposite hemisphere). The (sub)cluster field L-medial represents the ensemble of voxels within the most medial portion of the large Field L cluster. The zero level corresponds to the estimated mean during rest periods and the error bars to standard errors across voxels in the cluster. c, caudal; d, dorsal; r, rostral; v, ventral; NCM, caudomedial nidopallium (^*^p_FWE_ < 0.05; °*p*_FWE_ < 0.10; *n* = 15).

The statistical map of the main effect of stimulus class (Figure 3A) shows that song filtering (either temporal or spectral) affects the activation of primary (Field L) and secondary (caudomedial nidopallium, NCM) auditory forebrain regions in both hemispheres with the most distinct effect in the left hemisphere. The following *post-hoc* tests clarify the nature of the observed effect (Figures 3B–E). An overview of the supra-threshold clusters for this analysis is given in Table 1. The effect of temporal filtering was most pronounced in right Field L where a decrease in BOLD response to the filtered song compared to the response to non-manipulated song was observed (Figure 3B). In contrast, the effect of spectral filtering was most prominent in the left hemisphere with an increased response in both Field L and NCM after filtering (Figure 3C). In the right hemisphere, we observed a small cluster within Field L with decreased responses to spectrally filtered song vs. non-manipulated song. The strongest (in terms of *t*_*max*_) and most extended effect was observed when comparing the responses to the manipulated songs against each other. This effect was related to a significantly higher response to spectrally filtered song vs. temporally filtered song. Similar to the increased response of spectrally filtered as compared to non-manipulated song, this effect was mainly observed in left Field L and NCM.

**Table 1:** Summary of all supra-threshold clusters (*p*_uncorrected_ < 0.005) in the voxel-based analysis comparing BOLD responses to the different stimulus types.

**Contrast**	**Region**	**Hemisphere**	***F*_max/_*t*_max_**	**Extent (mm^3^)**
Main effect stimulus class	Field L + NCM	Left and Right	10.53	1.10
	Field L	Right	7.02	0.10
***Post-hoc t-*****tests**				
Non-manipulated song > Temporally filtered song	Field L (medial)	Right	3.59^*^	0.15
	Intersection Field L/NCM (medial)	Right	3.45^*^	0.10
	Field L	Left	2.64°	0.05
Temporally filtered song > Non-manipulated song	No supra-threshold clusters			
Non-manipulated song > Spectrally filtered song	Field L (medial)	Right	2.92°	0.05
Spectrally filtered song > Non-manipulated song	NCM	Left	4.05^*^	0.40
	Field L	Left	3.14^*^	0.15
Spectrally filtered song > Temporally filtered song	Field L + NCM	Left	4.37^*^	0.95
	Field L (medial)	Right	3.35^*^	0.10
	Intersection Field L/NCM (medial)	Right	3.42^*^	0.05
Temporally filtered song > Spectrally filtered song	No supra-threshold clusters			

One way ANOVA—within subjects (n = 15); For post-hoc t-tests:

* *p_FWE_ < 0.05; °p_FWE_ < 0.10*.

### Lateralization

**Figure 4:**
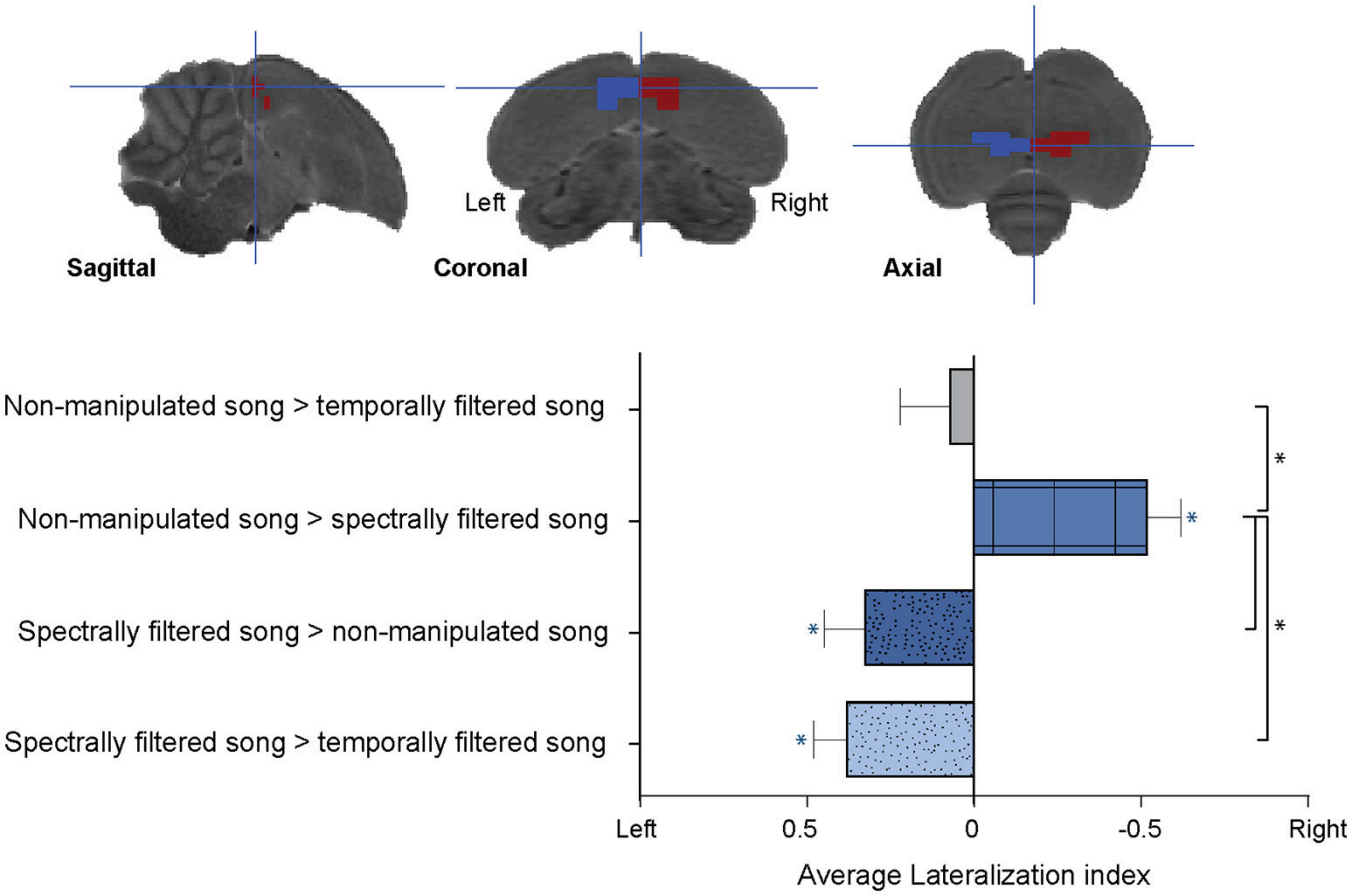
Lateralization of the sensitivity to spectral and temporal filtering. **(Top)** Illustration of the left and right regions of interest for the lateralization analysis overlaid on sagittal, coronal, and axial slices from the population based template. **(Bottom)** Graph indicating the average lateralization index for the different filtering effects. The error bars represent the standard error of the mean across subjects. The blue asterisks indicate a significant lateralization of the respective effect to either the left or right (lateralization index significantly different from zero) (^*^p < 0.05; *n* = 15).

Although the effect of spectral and temporal filtering was observed in both hemispheres, the relative pattern of activations showed a clear dissociation between hemispheres depending on the type of filtering. To study this further, we calculated LIs for each of the observed filtering effects (i.e., increased or decreased response to one stimulus type relative to responses elicited by the other two stimulus types) in each subject. The ROI for this analysis was composed of the ensemble of voxels (and their mirrored counterparts) in which a significant effect of song filtering was found in the above described voxel-based group analysis (Figure 4, Top).

We found a significant right lateralization for the decrease in BOLD response after spectral filtering of song (AveLI = −0.5218 ± 0.09818; *p* < 0.0001). In contrast, we observed a significant left sided bias for the increase in BOLD response to the spectrally filtered song relative to non-manipulated (AveLI = 0.3245 ± 0.1261; *p* = 0.0111) and temporally filtered song (AveLI = 0.3783 ± 0.1011; *p* = 0.0011). This observed left lateralization corroborated the increase in BOLD response revealed by the voxel-based analysis. In contrast to the lateralization observed for the effects of spectral filtering on the BOLD response, no significant lateralization was observed for the decrease in response after temporal filtering (AveLI = 0.0688 ± 0.1497; *p* = 0.3263; Figure 4, Bottom).

Direct comparison of the calculated LIs revealed a strong main effect of filtering effect [*F*_(3, 42)_ = 13.3502, *p* < 0.0001; Figure 4]. *Post-hoc* analysis indicated that the right sided bias observed for the decrease in BOLD response after spectral filtering was significantly different (*p* = 0.0033) from the decrease in neuronal response after temporal filtering, which was as stated above found to be more bilateral. Second, this right hemispheric bias for the decrease in BOLD response after spectral filtering was in strong contrast to the left hemispheric bias observed for the increase in BOLD response after similar (spectral) filtering of the song (LI spectrally filtered > non-manipulated song vs. LI non-manipulated song > spectrally filtered song: *p* < 0.0001; LI non-manipulated > spectrally filtered song vs. LI spectrally song > temporally filtered song: *p* < 0.0001). Note that an additional analysis was performed in which the large ROI was split into two separate ROIs including only Field L or NCM voxels, respectively. For both the Field L and NCM ROIs, this analysis yielded similar results as those reported for the large ROI with more pronounced effects in Field L as compared to NCM (Figure S3).

## Discussion

The auditory forebrain in songbirds is specialized to selectively process conspecific song (Grace et al., 2003; Hsu et al., 2004). Specific neuronal tuning to natural spectral and temporal modulations in song is thought to drive this selectivity and as such enable discrimination of natural sounds in the auditory system (Woolley et al., 2005). In the present study, we used fMRI to investigate the influence of filtering the natural spectral or temporal modulations from song, on BOLD responses in the auditory forebrain. It has been shown that responses at the population level in forebrain regions are indicative of response selectivity for song over other complex sounds (Grace et al., 2003). In line with this, we detected robust BOLD responses to song across auditory forebrain regions and found that these responses were influenced by our filtering procedures. Analyzing differences between the responses to the non-manipulated songs vs. the filtered songs revealed sensitivity to manipulations of the natural spectral and/or temporal song structure in both field L and NCM. Although the classical view on auditory processing dictates that neural coding of song becomes more specialized and complex in higher order auditory regions (Theunissen and Shaevitz, 2006), our results indicate equivalent population response selectivity in primary field L and secondary NCM. These findings confirm earlier observations by Grace and colleagues who found that, when considering average firing rate, selectivity for conspecific song vs. synthetic songs with changed spectro-temporal statistics, was similar in Field L and NCM (Grace et al., 2003).

The specific selectivity patterns for natural song vs. filtered song observed in the present study appeared particularly interesting. Spectral filtering induced the most pronounced changes in BOLD responses and showed distinct effects in the two hemispheres. We found that reducing natural spectral structure in conspecific song elicits an increase in BOLD signal in left hemispheric auditory forebrain structures and a subtle decrease in the right hemisphere. In contrast, a reduction of temporal information in conspecific song induced a small bilateral decrease in BOLD signal in Field L. Although we cannot exclude that filtering in one dimension (i.e., frequency or time) did not influence the other dimension to some extent, the fact that filtering of spectral modulations reduced right hemispheric neural activity suggests a right hemispheric selectivity to process spectral song information. This right hemispheric selectivity for spectral elements of song was also previously demonstrated when the lesioning of right hemispheric auditory circuitry reduced the ability of zebra finches to process harmonic structure in song (Cynx et al., 1992) and might be analogous to right hemispheric spectral domain processing observed in the human auditory cortex (Obleser et al., 2008). The strong increase in left hemispheric activity after spectral filtering is a novel finding which is discussed in greater detail below.

An earlier study from our group similarly analyzed fMRI data obtained by presenting zebra finches with natural conspecific song and spectrally and temporally filtered versions of the song (Boumans et al., 2007). In this study, the strongest effects were observed after modulation of temporal structure and no hemispheric asymmetries were detected (Boumans et al., 2007). We believe that the difference in relative response patterns for the different stimulus types compared to the present results may be due to considerable methodological differences between the two studies. This previous study employed a different pulse sequence and acquired only a single coronal slice through the songbird brain. This protocol limited the studied region to only the ventral part of the NCM, central field L, and the dorsal part of CMM. The present study assessed responses in the entire forebrain using an SE RARE sequence. The latter allows very accurate between-subject voxel-by-voxel comparisons of the BOLD responses to the different stimulus types. Such voxel-based statistics were not performed in the earlier study and responses in larger sub-regions were averaged to interpret results. Additionally, temporal filtering in this previous study was greater than in the present study such that the syllabic structure was removed altogether. Such stringent temporal filtering, which retained spectral content, may have produced spectrally refined stimuli that elicited stronger activity from a tonotopically organized area, such as Field L (Zaretsky and Konishi, 1976). Future studies of the hemispheric specialization in the zebra finch may be well-served to incorporate such stringent temporal filtering into their experimental designs to investigate this further. Further, although responses to filtered song under isoflurane are reported by Boumans and point to limited effects of filtering on the BOLD response, the final interpretation of the effects described was based on data acquired under medetomidine anesthesia. The discrepancy between results over the different studies might point to an important effect of anesthesia on response selectivity and lateralization of auditory processing as was observed earlier in starling (George et al., 2004, 2005). This finding implies that data on lateralization should be carefully interpreted and verified with data obtained in different states of wakefulness before being able to relate the selectivity of auditory forebrain regions found in this study to perceptual behavior.

The fact that the birds were anesthetized during fMRI in this study whereas humans are seldom anesthetized during studies on hemispheric lateralization of speech processing complicates any comparison between the present study and similar experiments in humans. However, studies reporting left hemispheric specialization for birdsong employed a variety of techniques including ZENK expression (Avey et al., 2005; Moorman et al., 2012), behavioral training (Cynx et al., 1992), and single unit recording (Hauber et al., 2007), only the last of which employed anesthesia of any kind. Likewise, a neuroimaging study of humans reported preserved left hemispheric dominance for receptive language (i.e., speech sounds) during general anesthesia (Rezaie et al., 2014). Nonetheless, employing neuroimaging protocols that require no sedation of the songbirds would be a worthy goal for future studies as this approach would simplify comparisons between human and avian fMRI studies.

The most pronounced effect observed in the current study, which has to our knowledge not yet been described earlier in any other songbird study, was the increase in left hemispheric activity after spectral filtering. Although such an effect has not been described in human speech processing, this finding is highly suggestive of a left hemispheric specialization for temporal domain processing that parallels what is commonly reported in humans (Efron, 1963; Schwartz and Tallal, 1980). Our spectral filtering retained much of the temporal content from the conspecific song, so the increase in left hemispheric activity likely reflects a form of temporal domain processing that is routinely suppressed by the spectral content of natural sounds. This specialization for temporal domain processing would appear to differ somewhat from that which is reported in humans. In humans, increasing mean formant transition rate, increasing temporal complexity, or removing spectral content tends to cause a drop in right hemispheric activity relative to activity in the more consistent left hemisphere (Belin et al., 1998; Zatorre and Belin, 2001; Schonwiesner et al., 2005; Obleser et al., 2008). Thus, a left hemispheric specialization for temporal domain processing that is analogous, but not homologous, to what is reported in humans might have developed in the zebra finch auditory forebrain. With this interpretation of the data, one would expect to also see effects of temporal filtering in the left hemisphere which was not observed in the present study. It is possible that this lack of a lateralized response to the reduction of temporal information, and the preservation of spectral information, may be due to the relatively conservative level of temporal filtering used to maintain a degree of syllabic structure and to avoid confounding serial order as outlined above. Thus, temporal sensitivity might be mainly driven by temporal information between syllables rather than by the fine temporal structure within the syllables. This should be verified by exposing zebra finches to song stimuli with different degrees of temporal and spectral filtering. Such analysis would also help in differentiating between effects on the BOLD response induced by the nature of filtering (temporal vs. spectral) vs. the magnitude of the filtering.

In summary, our results in zebra finches demonstrate that both primary (Field L) and secondary (NCM) auditory forebrain regions in the left and right hemispheres of zebra finches are sensitive to artificial manipulations of temporal and/or spectral structure in conspecific song. Additionally, we found that the hemispheres differ substantially in how they process these different types of information, pointing to an asymmetric sensitivity to spectral and temporal song structure in the auditory forebrain. The possible left hemispheric sensitivity to temporal structure, together with the observed right hemispheric sensitivity to spectral structure, suggest parallels between our asymmetry findings in the zebra finch forebrain and the classical view on spectral vs. temporal domain processing in the human auditory cortex (Schwartz and Tallal, 1980; Sidtis et al., 1981; Belin et al., 1998; Obleser et al., 2008). Although more studies are necessary to confirm this, our results already provide a strong indication that songbirds can possibly represent a valuable animal model for studying the neural basis of hemispheric asymmetry in auditory processing of complex, learned conspecific communication sounds.

## Author contributions

LV and SW contributed equally to this work. Conceived and designed the experiments: LV, SW, GK, and AV; Performed the experiments: LV and SW; Performed final data analysis: LV; Advised on data analysis: JH, MV, and GK; Contributed reagents, materials and/or analysis tools: AV; Wrote the paper: LV and SW. All authors critically reviewed the manuscript and approved the final version.

### Conflict of interest statement

The authors declare that the research was conducted in the absence of any commercial or financial relationships that could be construed as a potential conflict of interest.
