# Supplementary material for: Song Processing in the Zebra Finch Auditory Forebrain Reflects Asymmetric Sensitivity to Temporal and Spectral Structure
Source: Front Neurosci. 2017 Oct 5;11:549. doi: 10.3389/fnins.2017.00549 (PMC5633600; doi:10.3389/fnins.2017.00549)
Supplement: Supplementary file 13 [file DataSheet1.PDF]

Supplementary Material

Song Processing in the Zebra Finch Auditory Forebrain Reflects Asymmetric Sensitivity to Temporal and Spectral Structure

Lisbeth Van Ruijssevelt[1], Stuart D. Washington[1], Julie Hamaide[1], Marleen Verhoye[1], Georgios A. Keliris[1], and Annemie Van der Linden[1]*

[1] Bio-Imaging lab, Department of Biomedical Sciences, University of Antwerp, Antwerp, Belgium

*** Correspondence:** Corresponding author **-** Annemie.VanderLinden@uantwerpen.be

1 Supplementary Figures

(see next page)

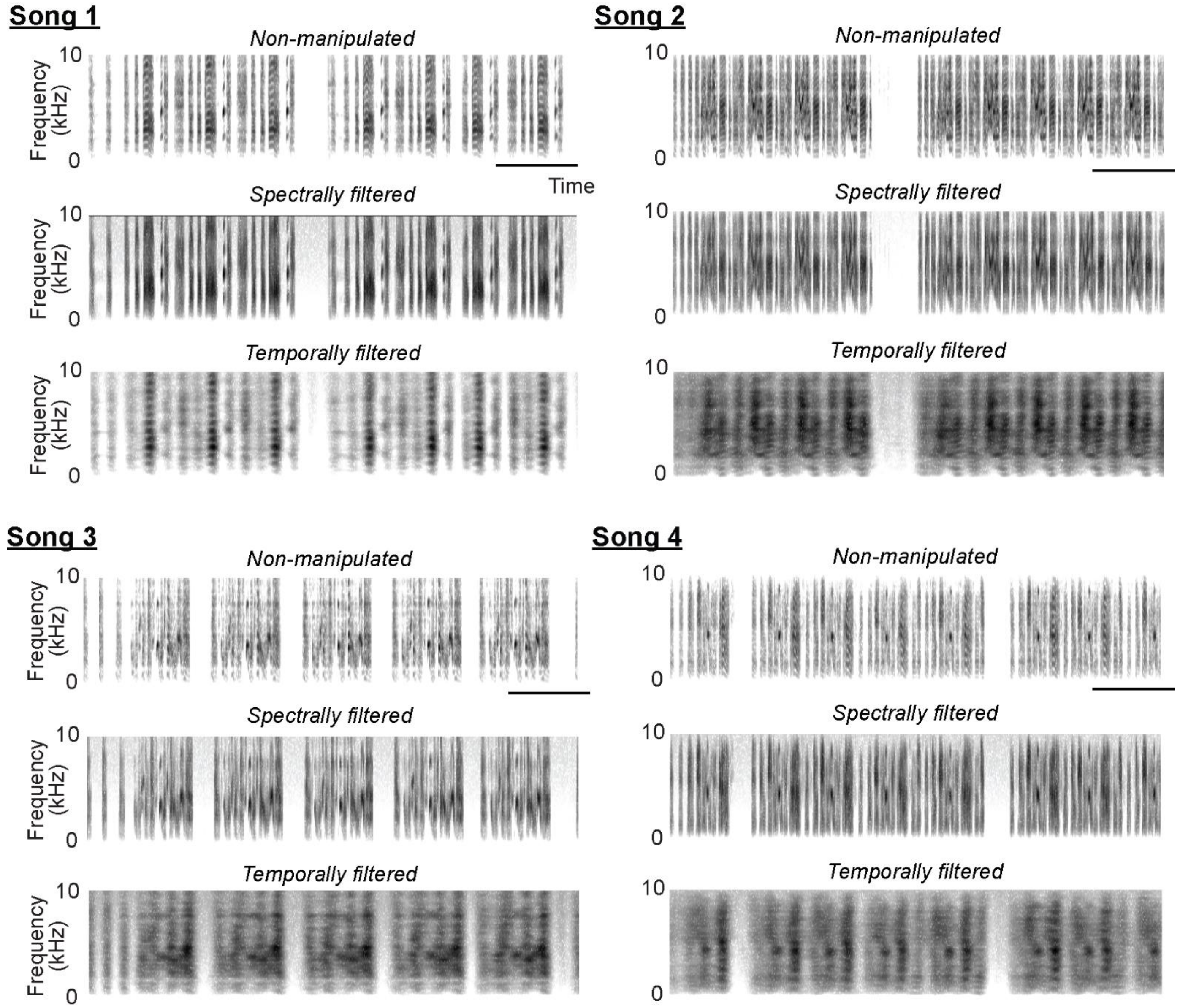

Supplementary Figure 1. Spectrograms of the different songs and their filtered versions used as stimuli in the fMRI experiment. Scale bar = 1s.

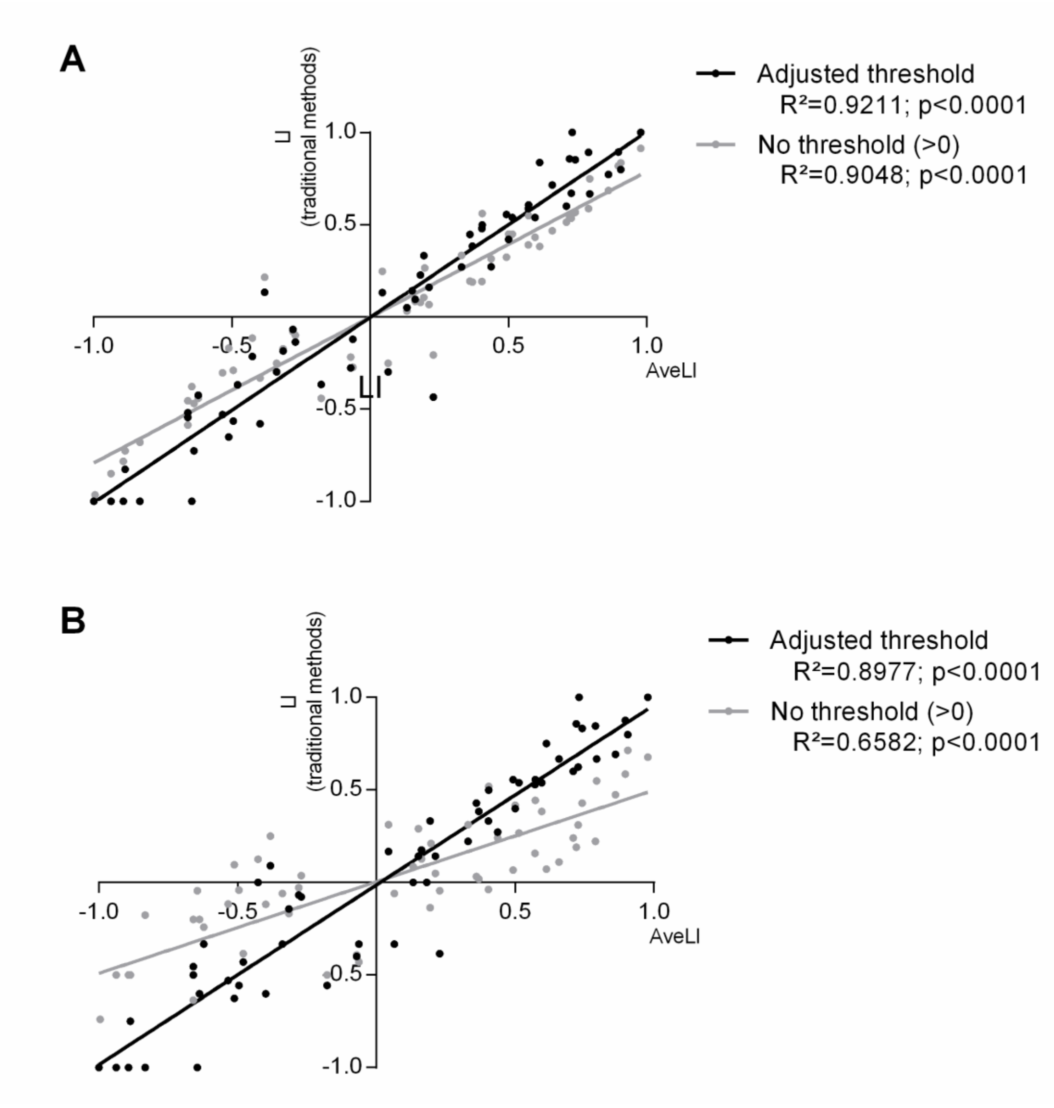

Supplementary Figure 2. Regression analysis of lateralization indices (LIs) calculated using AveLI versus alternative (more conventional) methods [1]. The LIs for the different methods show a strong correlation suggesting that our results were not significantly affected by the method chosen to calculate LIs. (A) Correlation between AveLIs and LIs that were calculated based on the magnitude of the signal. The grey datapoints and trendline correspond to LIs calculated using the sum of the positive t-values including all positive voxels (t>0) in the left versus the right ROI. The black data points and trend line correspond to LIs calculated using the sum of the positive t-values including only voxels exceeding a subject specific adjusted threshold which was calculated based on the following procedure: First the mean of the 5% most activated voxels within the left and right ROI was calculated. Second, the threshold was determined corresponding to 50% of this mean [2]. (B) Correlation between AveLIs and LIs which were calculated based on the number of activated voxels. The grey datapoints and trendline correspond to LIs calculated based on the number of positive voxels (t>0, no threshold). The black data points and trend line correspond to LIs calculated based on the number of significant voxels determined by the subject specific threshold as described in A.

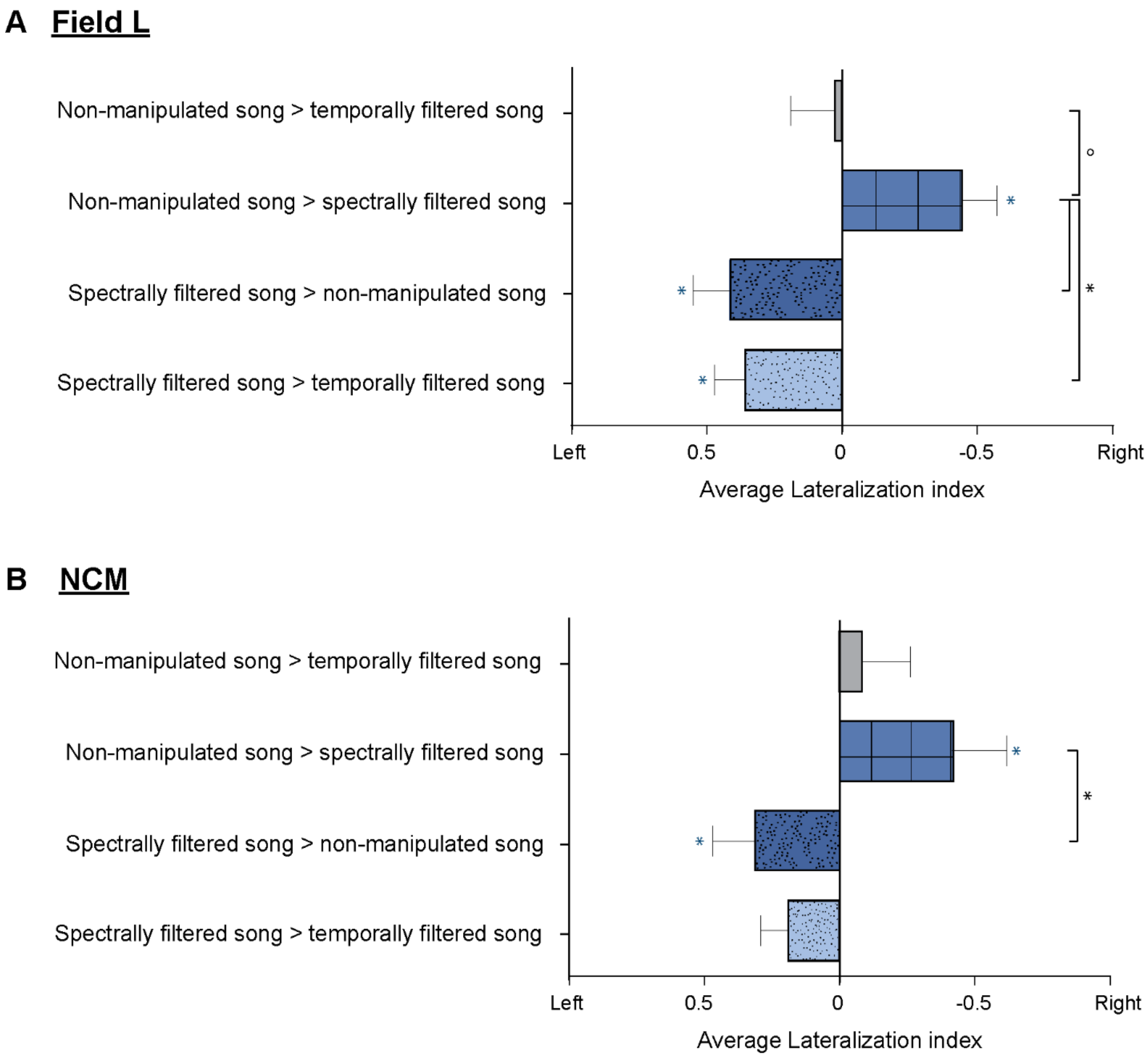

Supplementary Figure 3. Lateralization of the sensitivity to spectral and temporal filtering in Field L (A) and NCM (B). The Field L and NCM ROIs used in this analysis included the significant voxels and their mirrored counterparts from the main effect of stimulus class (Fig 4A) which fell within the borders of the respective sub-regions (see delineations in Fig 3A). The graphs indicate the average lateralization index (LI) for the different filtering effects. The error bars represent the standard error of the mean across subjects. The blue asterisks point to a significant lateralization (LI>0; one sample t-test) of the respective effect to either the left or right. (*p<0.05; n=15)

2 Supplementary Audio files

Audio 1: Song 1, original / non-manipulated song stimulus

Audio 2: Song 1, spectrally filtered

Audio 3: Song 1, temporally filtered

Audio 4: Song 2, original / non-manipulated song stimulus

Audio 5: Song 2, spectrally filtered

Audio 6: Song 2, temporally filtered

Audio 7: Song 3, original / non-manipulated song stimulus

Audio 8: Song 3, spectrally filtered

Audio 9: Song 3, temporally filtered

Audio 10: Song 4, original / non-manipulated song stimulus

Audio 11: Song 4, spectrally filtered

Audio 12: Song 4, temporally filtered

3 References:

[1] M.L. Seghier, Laterality index in functional MRI: methodological issues. Magn. Reson. Imaging 26 (2008) 594-601.

[2] G. Fernández, A. de Greiff, J. von Oertzen, M. Reuber, S. Lun, P. Klaver, J. Ruhlmann, J. Reul, and C.E. Elger, Language Mapping in Less Than 15 Minutes: Real-Time Functional MRI during Routine Clinical Investigation. Neuroimage 14 (2001) 585-594.
